# The novel aminoglycoside, ELX-02, permits *CTNS^W138X^* translational read-through and restores lysosomal cystine efflux in cystinosis

**DOI:** 10.1371/journal.pone.0223954

**Published:** 2019-12-04

**Authors:** Emma J. Brasell, Lee Lee Chu, Murielle M. Akpa, Idit Eshkar-Oren, Iris Alroy, Rachel Corsini, Brian M. Gilfix, Yojiro Yamanaka, Pedro Huertas, Paul Goodyer

**Affiliations:** 1 McGill University, Department of Human Genetics, Montreal, Canada; 2 Research Institute of the McGill University Health Centre, Montreal, Canada; 3 McGill University, Department of Experimental Medicine, Montreal, Canada; 4 Montreal Children’s Hospital, Department of Nephrology, Montreal, Canada; 5 Eloxx Pharmaceuticals, Inc., Waltham, United States of America; University Medical Center Utrecht, NETHERLANDS

## Abstract

**Background:**

Cystinosis is a rare disorder caused by recessive mutations of the *CTNS* gene. Current therapy decreases cystine accumulation, thus slowing organ deterioration without reversing renal Fanconi syndrome or preventing eventual need for a kidney transplant.15-20% of cystinosis patients harbour at least one nonsense mutation in *CTNS*, leading to premature end of translation of the transcript. Aminoglycosides have been shown to permit translational read-through but have high toxicity level, especially in the kidney and inner ear. ELX-02, a modified aminoglycoside, retains it read-through ability without the toxicity.

**Methods and findings:**

We ascertained the toxicity of ELX-02 in cells and in mice as well as the effect of ELX-02 on translational read-through of nonsense mutations in cystinotic mice and human cells. ELX-02 was not toxic *in vitro* or *in vivo*, and permitted read-through of nonsense mutations in cystinotic mice and human cells.

**Conclusions:**

ELX-02 has translational read-through activity and produces a functional CTNS protein, as evidenced by reduced cystine accumulation. This reduction is comparable to cysteamine treatment. ELX-02 accumulates in the kidney but neither cytotoxicity nor nephrotoxicity was observed.

## Introduction

Cystinosis is a rare autosomal recessive disorder caused by mutations of the *CTNS* gene, encoding a cystine-specific transporter (cystinosin) that facilitates cystine efflux through the lysosomal membrane [1]. Homozygous *CTNS* mutations cause intralysosomal cystine accumulation, disturbance of cellular homeostatic mechanisms and progressive organ dysfunction. At birth, babies with biallelic null *CTNS* mutations appear normal. However, between 4–6 months of age, they develop severe proximal tubular dysfunction (renal Fanconi Syndrome) and failure to thrive. By 12–14 months of age, microdissection studies show atresia of the proximal tubule (swan-neck lesion). Between 5–10 years of age, there is progressive glomerular podocyte injury, proteinuria and renal insufficiency; dialysis is required by 10–12 years of age [2, 3]. Although kidney transplantation resolves renal insufficiency, inexorable deterioration of other organs slowly leads to hypothyroidism, diabetes, brain dysfunction and profound muscle wasting that compromises respiration and swallowing. Without further treatment, life expectancy is less than 30 years.

In the 1970’s, Thoene *et al*. discovered that pathologic intralysosomal accumulation of cystine in mutant cells could be dramatically decreased by the reducing agent, cysteamine [4]. Oral cysteamine (1.3g/m^2^/day) reduces leukocyte cystine content to about 15–20% of baseline and, when therapy is initiated by 24 months, the drug slows deterioration of the kidneys and other organs [5, 6]. However, while oral cysteamine therapy has substantially altered the natural history of cystinosis, it does not reverse the renal Fanconi syndrome and most patients eventually require renal transplantation. Muscle wasting remains a significant complication during the third decade and longevity remains compromised. In part, the suboptimal benefit of cysteamine has been attributed to its poor tolerability and there is hope that the advent of a delayed release form will be helpful in older children and adults. On the other hand, CTNS protein (cystinosin) may have cellular functions beyond its role as a cystine-selective channel in the lysosomal membrane. *CTNS* mutant proximal tubular cells exhibit defective megalin-dependent endocytosis of luminal proteins *in vitro* and this is refractory to cysteamine treatment [7]. Furthermore, a CTNS isoform (CTNS^LKG^) constituting 10–15% of total cell cystinosin is expressed at the luminal membrane suggesting an alternative function [8]. Other studies show that the CTNS protein is required for vesicular traffic involved in autophagy [9]. Thus, successful therapy of cystinosis may require a complementary strategy to offset the defect in these non-channel functions.

Worldwide, the most common cystinosis mutation is a 57 Kb deletion that eliminates the first 10 exons of the gene and is thought to have arisen in Northern Europe in about 500 AD [10]. In Canada, however, the most common mutant allele is a nonsense mutation (*CTNS*^*W138X*^), introduced from Ireland into the French Canadian population [11]. The W138X mutation creates a premature STOP codon in the seventh exon of the *CTNS* gene and accounts for 40–50% of cystinosis alleles in Quebec [11]. Interestingly, Heier and DiDonato reported in 2009 that premature STOP codons can be overcome by aminoglycoside antibiotics (eg. geneticin) [12]. These drugs bind to the mammalian ribosome, relax translational fidelity and allow read-through of premature STOP codons which would otherwise bring translation to a halt and induce transcriptional decay [13, 14]. Unfortunately, this observation has not been translated into a useful clinical therapy because of the inherent renal and cochlear toxicity of aminoglycosides in humans. It is conceivable that cochlear toxicity is linked to an interaction between aminoglycosides and the mitochondrial ribosome which is homologous to the ribosome of prokaryotes. Thus, the antibacterial effects of aminoglycosides are accompanied by disturbance of mitochondrial protein synthesis in humans. Individuals with specific mitochondrial genetic variants that alter the mitochondrial ribosome are particularly susceptible to aminoglycoside-induced renal injury and cochlear dysfunction [14].

**Fig 1:**
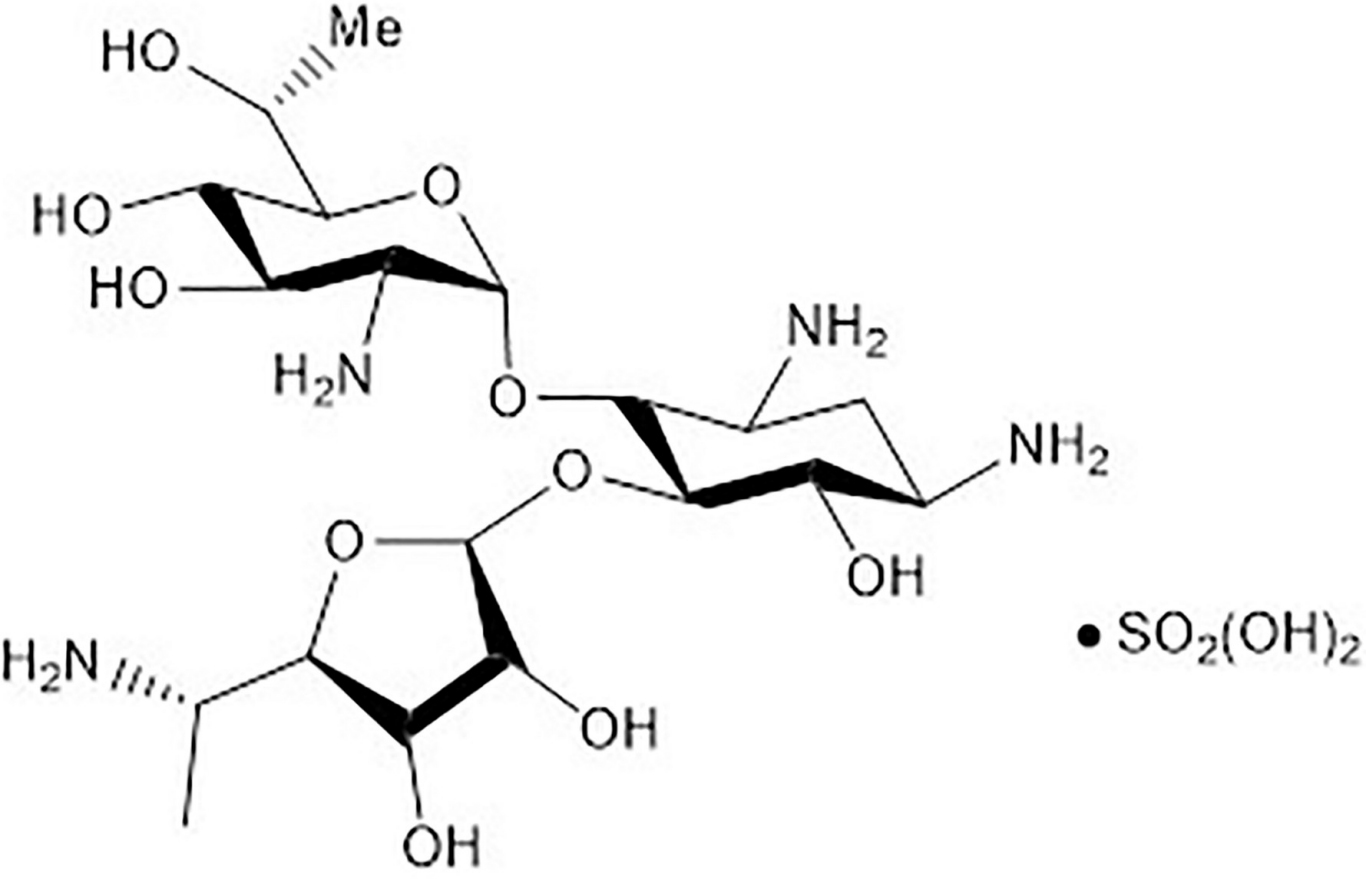
ELX-02 structure. Molecular structure of the ELX-02 compound.

To overcome this obstacle, Eloxx Pharmaceuticals recently developed a series of Eukaryotic Ribosomal Specific Glycosides (ERSG’s) which were iteratively screened for translational read-through of premature STOP codons with diminished binding to the bacterial (and thus mitochondrial) ribosome. A fifth-generation compound, ELX-02 (Fig 1), showed poor bactericidal activity (>100X increase in mean inhibitory concentration, MIC, for E coli), weak inhibition of mitochondrial protein synthesis (50X increase in mitochondrial inhibitory concentration, IC50Mit) and 10-20X reduction in toxicity (lethal concentration, LC50) for HeLa cells (Table 1).

**Table 1:** Comparison of ELX-02 to Gentamicin and G418.

**Toxic ribosomal effects of aminoglycoside**				
	Gentamicin		G418	ELX-02
Antibacterial activity MIC (mM)	6		9	680
Mitochondria IC_50_^Mit^ (mM)	26 ± 2		13 ± 1	965 ± 155
Cell toxicity LC_50_ (mM)	2.5 ± 0.3		1.3 ± 0.1	22.2 ± 1.1
**Readthrough effect of aminoglycoside**				
	Mutation	Gentamicin	G418	ELX-02
Usher syndrome	R3X	0.1	17	22
Usher syndrome	R245X	0.3	2.2	2.1
Hurler syndrome	Q70X	0.2	4.2	4.5
Cystic fibrosis	G542X	0.5	6	6

Importantly, 7.5 μM ELX-02 induced 2–22% translational *in vitro* read-through for a variety of nonsense mutations associated with Usher Syndrome, Hurler Syndrome and Cystic Fibrosis. ELX-02 read-through was increased (6–22 times) that of gentamycin (Table 1) [15, 16].

In this study, we explore the potential of ELX-02 to serve as a novel therapy for cystinosis caused by *CTNS* nonsense mutations. In fibroblasts from cystinosis patients, we report that ELX-02 permits translational read-through of the *CTNS*^*W138X*^ nonsense mutation without overt cellular toxicity. Furthermore, we show that CTNS protein expression (and its attendant correction of nonsense mutation-mediated transcript decay) is sufficient to reverse pathologic intralysosomal cystine accumulation. In a novel *Ctns*^*Y226X*^ nonsense mutant mouse, we demonstrate that subcutaneous ELX-02 accumulates in kidney tissue without overt renal toxicity and that ELX-02 (10mg/kg X2/week for 3 weeks) reduces renal cystine accumulation *in vivo*.

## Materials and methods

### Cell culture

The human proximal tubule cell line HK-2 (American Type Culture Collection, USA) was maintained as adherent monolayer cultures in DMEM-F12 culture medium (Life Technologies, USA), supplemented with 10% fetal bovine serum (Life Technologies, USA), 1% glutamine and Penicillin-Streptomycin (Life Technologies, USA), 1x Insulin-Transferrin-Selenium (Corning, USA), 36 ng/mL hydrocortisone(Sigma, USA), 10 ng/mL epithelial growth factor (Tonbo Biosciences, USA), and 40 pg/mL tri-iodothyronine (Sigma, USA). HEK293 (American Type Culture Collection, USA) cell line was maintained in DMEM (Life Technologies, USA) supplemented with 10% FBS, 1% glutamine and 1% Penicillin-Streptomycin. Normal fibroblast cell line MCH070 and cystinotic fibroblasts cell lines WG0881, WG1012 and WG2379 were obtained from the Hereditary Renal Disease Cell Repository from the RI-MUHC (IRB protocol # 2018–2922) and maintained as monolayer cultures growing in DMEM (Corning, USA) supplemented with 15% fetal bovine serum. Cells were incubated at 37°C in an atmosphere of 5% CO_2_. Experiments were performed when cells reached 70–80% confluence. Trypsinization was performed using 0.25% trypsin, 2.21 mM EDTA (Corning, USA), cells were washed 3 times with PBS (Life Technologies, USA) then pellets snap frozen in an ethanol bath. Samples were store at -80°C until preparation for mRNA, protein or half-cystine analysis.

### Cytotoxicity assay

A Cell Counting Kit-8 was obtained from Enzo Life Sciences (Farmingdale, NY). Culture media, fetal bovine serum, and antibiotics were purchased from Invitrogen (Carlsbad, CA). Human Proximal tubule cells (HK-2), Wild type human fibroblast cells (MCH070) and human Fibroblast with W138X homozygous mutation (WG1012) were cultured in DMEM supplemented with 10% fetal bovine serum (FBS). Cells were trypsinized and re-suspended in fresh media at a concentration of 300,000 cells/well in 24-well plates. Cells were allowed to grow overnight at 37°C in a humidified 5% CO2 environment. G418 and ELX-02 were added to final concentration 0, 100 and 400 μg/mL. Following a 48–72 hours exposure to the drug, 50 μL of the cell counting reagent was added directly to the cell cultures in 24-well plates that have 500 μL of media. Cells were incubated in a humidified environment at 37°C, 5% CO2 for 1 h. After incubation, the absorbance at 450 nm was determined using Epoch Microplate reader (BioTek Instruments). The following equation was used to calculate the percentage of cell viability: cell viability (%) = (OD sample_(mean)_)/ (OD control_(mean)_) x 100, where OD is the optical density; OD of sample: mean absorbance of treated cells and OD of control: mean absorbance of control cells. To determine the percentage cytotoxicity, calculate the average absorbance of the triplicate samples and controls, the values for each concentration were converted into percentage of control (interpreted as 100% viability).

### Plasmid construction

The generation of the pcDNA3.1-*CTNS*, pcDNA3.1-*CTNS-DsRed* and the pcDNA3.1-*CTNS*^*W138X*^*-DsRed* plasmids has been previously described [17]. To generate carboxyl-terminal histidine (HIS)-tagged pcDNA3.1-*CTNS-His*, the plasmid *CTNS-His*-(1280–1697) plasmid was constructed by PCR, utilizing primers hCTNSmsc(F) 5’-GGTGGCCAGCGCGTGTC-3’′; *Msc1* site underlined, hCTNS-his(R) 5’GCCTCGAGCTAGTGGTGGTGGTGGTGGTGGTTCAGCTGGTCATACCCCGG-3’ and hCTNSLKG-his(R) 5’GCCTCGAGCTAGTGGTGGTGGTGGTGGTGGCCCTTCAAGCTGCTTGCAGAAAC-3’′; *Xho1* site underlined. The PCR product was digested with Msc1 and *Xho*I and cloned into the same sites of pcDNA3.1-CTNS. To generate amino-terminal kozak pcDNA3.1-*CTNS-His*, the kozak-*CTNS-His*-(594–1280) plasmid was constructed by PCR, utilizing primers kozakctns1 5’GCTCGGATCC**GCCGCCACCATG**ATAAGGAATTGGCTGACTATTTTTATC-3’′; *BamH1* site underlined and kozak sequence in bold, hCTNS-msc (R) 5’GCTGGCCACCGCGCTCATAC-3’′; *Msc1* site underlined. The PCR product was digested with BamH1 and Msc1 and cloned into the same sites of pcDNA3.1-*CTNS-His*. Plasmid pcDNA3.1-kozak-*CTNS*^*W138X*^*-His* containing the UGAW138X was constructed by PCR-mediated mutagenesis with primers kozakctns1 and hCTNSbsu361(R) 5′-CACCTGAGGGTAGAAGGAGATGGATCAGGCCAC-3′ (W138X mutation site underlined). The PCR products were ligated into the plasmid pcDNA3.1-kozak-*CTNS*^*W138X*^*-His*.

### Transfection and ELX-02 treatment for read-through assay

The transfection of the pcDNA3.1-*CTNS-DsRed*, pcDNA3.1-*CTNS*^*W138X*^*-DsRed*, and pcDNA3.1-kozak-*CTNS*^*W138X*^*-His* plasmids has been previously described [17]. In experiments where an aminoglycoside was used to induce ribosomal read-through, 0–400 μg/mL of ELX-02 (Eloxx Pharmaceuticals, Israel) or G418 (Life Technologies, USA) were added to the culture flasks after 48 h of transfection. Aminoglycosides were dissolved directly at its final concentration in DMEM. Control experiments were performed using cells exposed to the identical media without aminoglycosides. Then the cells were cultured in DMEM media containing 0–400 μg/mL of aminoglycosides for 48 h. Cell pellets were washed and assayed for RNA, protein and half-cystine content as previously described [17]. For the read-through assay in cystinotic fibroblast cells, triplicates of each fibroblast cell lines were seeded in T25 culture flask and grown to confluency. Then the cells were washed with 1x PBS three times and cultured in DMEM media containing 20–400 μg/mL aminoglycosides and/ or 50 μM cysteamine for 24–72 h. Cell pellets were washed and assayed for RNA, protein and half-cystine content as previously described [17].

### Confocal microscopy

After aminoglycoside treatment, HEK293 cells transfected with pcDNA3.1-*CTNS-DsRed*, pcDNA3.1-*CTNS*^*W138X*^*-DsRed* were seeded in 4-well-chambered coverglass (Nunc) and fixed in 4% paraformaldehyde for 10 min at RT. After washing two times with PBS, the cells were quenched with 20mM ammonia chloride for 10 minutes and wash with PBS. Then the cells were blocked (PBS, 5% normal goat serum, 0.1% Triton X-100, 1% BSA) for 1 h, at RT. After triple washing with PBS, the cells were stained with DAPI and mounted onto slides with ProLong Gold antifade reagent (Life Technologies). The cells were observed using a Zeiss LSM780 laser scanning confocal microscope.

### Western immunoblotting

Urine was collected from mice in opaque microfuge tubes, centrifuge at 15 000 rpm for 10 min at 4°C and stored at -80°C until ready to use. 30 μL of urine was assayed via SDS-PAGE followed by standard immunoblotting method and analysis. Primary antibody overnight incubation with anti-Transferrin (GeneTex, USA), anti-RBP4 (Adipogen, USA) and anti-B2M (GeneTex, USA) at 1:1000 ratio was followed by secondary antibody incubation for 1h with α-rabbit IgG-HRP (Life Technologies, USA) or α-mouse IgG-HRP (Life Technologies, USA). Bands were visualized using ECL 2 Western Blotting Substrate (Thermo Scientific Pierce, USA).

### RNA extraction and real-time PCR

Total RNA extraction and real-time PCR has been previously described [17].

### Intracellular half-cystine measurement

This assay is a derivative of that of Gilfix *et al*. [18]. *Stock solutions of homocystine and* cystine were prepared assuming a assuming a molar absorption coefficient of 397 at 244 nm. This is the molar absorption coefficient of the disulfide bond (personal communication from D. Jacobsen, Cleveland Clinic Foundation). Stock solutions of cysteinylglycine were prepared on the basis of weight. The linear range for this assay is: 2–300 μmol/L L-homocysteine, 3–600 μmol/L L-cysteine, and 10–100 μmol/L cysteinylglycine. The within-run CV for cysteine at 3 μmol/L was 1.6% (n = 4). Prior to analysis, samples were supplemented to 30 μmol/L L-homocysteine as a second internal standard to normalize the cystine values. This was done as in some cases cystamine had been added to the cell cultures and this interfered with the measurement of the usual internal standard, cystamine. An aqueous solution of 10% tris(carboxyethyl) phosphine•HCl (Pierce Chemical Co.) (9 μL) was mixed with 5 μL of 100 μmol/L cystamine. This was added to 90 μL of the sample and mixed immediately. Following incubation for 30 min at room temperature, 90 μL of 10%TCA-1 mmol/L EDTA was added. The sample was centrifuged for 10 min at 15,600 x g and the supernatant recovered. The supernatant (100 μL) was then added to a separate tube containing 20 μL of 1.55 mol/L NaOH and 250 μL of 0.125 mol/L borate buffer-4 mmol/L EDTA, pH 9.5 and mixed immediately. To this, 100 μL ammonium 7-fluorobenzo-2-oxa-1,3-diazole-4-sulfonate (Wako Chemicals USA, Inc.) (1 mg/mL in 0.125 mol/Lborate buffer, pH 9.5) was then added and mixed immediately. The mixture was incubated for 1 h at 60°C. An aliquot (50 μL) was loaded onto a Hypersil 5 μ C18 ODS (250 x 4.6 mm) column run at 1.5 mL/min. The run buffer was 0.05 mol/L KH2PO4, pH 2.1 containing 4% acetonitrile and the fluorescence monitored (λ excitation = 385 nm; λ emission = 515 nm). The results representing the sum of cysteine, cystine and other mixed form of cysteine in the sample expressed as half-cystine (cysteine). Total protein was measured using the BCA Protein Assay kit (Biovision), as per manufacturer’s instructions (96-well plate format). Half-cystine results were corrected to total protein. To estimate pathologic intralysosomal cystine, we subtracted basal cell cystine (15.95 ± 1.44 nmol half-cystine/mg protein). Data was processed to give arithmetic mean for half-cystine levels.

### Non-radioactive pulse-chase assay

The Click-iT AHA (L-azidohomoalaine) for newly synthesized protein synthesis kit (Life Technologies, USA) was used for these studies and experiments were performed according to manufacturer’s recommendation. HK-2 cells were seeded in T75 flask to reach 80% confluent. Methionine in cells was depleted by culturing in methionine-free DMEM for 1 h. Then 50 μM of AHA (a methionine analog) was added into medium for 1 h. Cells were washed and then incubated with the complete medium. The cells were chased for indicated time. Subsequently, Harvested cells at different time points were lysed and AHA incorporated protein was biotinylated using Click-IT-DIBO Biotin (Life Technologies, USA). Proteins were then immunoprecipitated with Pierce High Capacity Streptavidin slurry agarose (Life Technologies, USA) and further analyzed by Western immunoblotting.

### Transgenic mice

All animal experiments were approved by the McGill University Facility Animal Care Committee (FACC). Wildtype C57Bl6 mice were purchased from Charles River Laboratories. A Zinc-finger nuclease (ZFN) pair targeting CTNS exon 8 was identified. We designed a template with homology arms, a premature STOP codon replacing amino acid 226 and a silent XbaI restriction site. The ZFNs and the template were injected into the pronuclei of zygotes that were transferred to pseudo-pregnant females. Two pregnancies were brought to term. Viable F0 pups with a heterozygous *CTNS*^*Y226X*^ allele were crossed to CD1 wildtypes to yield F1 progeny for the same *CTNS*^*Y226X*^ mutation. Heterozygous F1 littermates were interbred to produce multiple litters of F2 animals, in which germline transmission of *CTNS*^*Y226X*^ was confirmed by Sanger sequencing. F2 *CTNS*^*Y226X*^ heterozygotes were interbred to generate 4 litters of F3 homozygous *CTNS*^*Y226X/Y226X*^ mice. Genotyping was performed at weaning, using ear punch samples taken under aseptic conditions. For subsequent experiments, the animals were sacrificed using CO_2_ asphyxiation under isoflurane anesthesia, followed by pneumothorax, according to standard operating procedures. Material acquired for genotyping was done according to standard operating procedures by ear punching. Mice were housed in groups, in solid–bottom cages with contact bedding and nesting material. Animals were monitored daily by a veterinarian, checking for excessive grooming, fighting, wounds, lethargy, activity and respiratory rate. Cages were changed daily, and animals had freely available food and water. Sick or distressed animals were euthanized and excluded from the study. (FACC protocol # 2011–6026).

### Histology

Kidneys were obtained from 6-month old mice, processed and paraffin embedded. The kidneys were sectioned (7μm) and stained with hematoxylin and eosin according to standard protocol. Kidneys were obtained from 6-month old mice and processed as to not dissolve the crystals. The kidneys were sectioned and stained with toluidine blue, and visualized under polarized light [19].

### Creatinine assay

Urine was collected from mice in opaque microfuge tubes over the course of the experiment and at the time of sacrifice, centrifuged at 15 000 rpm for 10 min at 4°C and stored at -80°C until ready to use. Urine was diluted in 0.9% saline (1:10 ratio) and assayed using the Mouse Creatinine Assay Kit (Crystal Chem, USA) following the manufacturer’s instructions.

### Pharmacokinetics (PK)

Male and female 5–7 month old *Ctns*^*Y226X/Y226X*^ mice (n = 28) were allocated to the following treatment groups. In Group 1, twelve (n = 12) *Ctns*^*Y226X/Y226X*^ mice (6 animals/sex) received a single subcutaneous injection of ELX-02 at 10 mg/kg (free base) at dose volume of 5 mL/kg. Blood and kidney tissues were collected for quantification of ELX-02. Blood was collected at the following time points: 0.25, 0.5, 1, 2, 4 and 8 h post-dose. Kidneys were collected at the following time points: 0.5, 2, 4 and 8 h post-dose (1 animal/sex for each time point). In Group 2 sixteen (n = 16) *Ctns*^*Y226X/Y226X*^ mice were dosed twice weekly with either ELX-02 (10 mg/kg/dose, free base) or saline for 21 days (total of 7 administrations). Blood was collected after the last dose at the following time points: pre-dose, 0.25, 0.5, 1, 2, 4 and 8 h post-dose. Kidneys were collected at the following time points: pre-dose, 0.5, 2, 4 and 8 h post-dose (1 animal/sex for each timepoint). The collected blood and kidneys were used for quantification of ELX-02 levels, and quantification of half-cystine levels in kidney.

### Pharmacodynamics (PD)

Twenty-nine (n = 29) *Ctns*^*Y226X/Y226X*^ mice 5–7 month old (4–5 animals/sex/group) were randomized to receive one of the following treatments: i. ELX-02 30 mg/kg/dose (free base); ii. ELX-02 10 mg/kg/dose (free base); iii. Vehicle (0.9% NaCl). In addition, nine (n = 9) age-matched CD-1 control mice (4–5 animals/sex) were administered with vehicle (0.9% NaCl). The animals were dosed with ELX-02 or vehicle by subcutaneous injection, at dose volume of 5 mL/kg, twice weekly for a period of 28 days (total of 8 doses). At the end of treatment period selected tissues, including blood, brain, spleen, heart, kidney, liver, lungs, and cochlea were collected for quantification of ELX-02 levels.

### Plasma and tissue processing

After the animals were anaesthetized, blood samples were collected via cardiac puncture into tubes containing anticoagulant (K_3_EDTA), placed in crushed wet-ice and centrifuged (2000 g at 4 °C for 10 minutes). The resultant plasma was separated from the erythrocyte pellet and then transferred to uniquely labelled clear polypropylene tubes and frozen immediately at -80 °C until analysis.

Brain, spleen, heart, kidney, liver, lungs, and cochlea tissues were dissected as follows: once the animal was deeply anaesthetized, the skin was removed, the thorax opened, and the heart exposed. A 5 mL syringe with a 25G needle was inserted into the left ventricle, saline infused to flush any blood out of the organs at a flow rate 10 mL/min, infusion time 0.5 min, and infusion volume 5 mL. An infusion pump, containing saline, was switched on to prime the tubing and needle. The needle, with saline flowing, was inserted into the left ventricle, and saline pumped at flow rate 6.5–8 mL/min to flush any blood out of the tissues. A small incision was made into the right atrium, to allow any blood and /or saline to be released. Once the liquid coming out of the right ventricle was clear, the needle was removed, and the infusion pump switched off. The tissues were then removed and frozen at -80°C until analysis.

### Quantification of ELX-02 in plasma and tissue

Plasma samples were spiked with ELX-02 internal standard (ELX-02-13C, *d*3), processed by protein precipitation extraction, and analyzed by HPLC-MS/MS using a TurboIonspray interface with positive ion multiple reaction monitoring. The method was qualified over the range 10 to 10000 ng/mL with a lower limit of quantification of 10 ng/mL using a 25μL sample. ELX-02 concentrations in plasma were expressed as ng/mL.

ELX-02 was extracted from mouse tissue’ homogenate by solid phase extraction after addition of (ELX-02-13C, *d*3), and analyzed by LC-MS/MS using a TurboIonspray interface with positive ion multiple reaction monitoring. The method was qualified over the range 5 to 5000 ng/mL with a lower limit of quantification of 5 ng/mL using a 50 μL aliquot of mouse tissue homogenate. ELX-02 concentrations in tissue samples were expressed as ng/g.

### PK analysis

Plasma and kidney ELX-02 concentration-time data were analysed by non-compartmental analysis using Phoenix WinNonlin. The following parameters for ELX-02 were determined where feasible, using the linear-logarithmic trapezoidal rule:

Cmax = the maximum plasma concentration
Tmax = the time to reach Cmax
t½ = apparent terminal elimination half-life
Tlast = the last quantifiable time point
AUC0-t = the area under the plasma concentration time curve (AUC) from the start of dosing to Tlast

### Mouse embryonic fibroblasts

7–10 embryos from each *Ctns* and *Ctns*^*Y226X/Y226X*^ mice were obtained at 14–16 days of pregnancy. Liver, heart and blood were removed. The embryos were minced and treated with 0.25% Trypsin-EDTA overnight at 4°C. The cells were washed, resuspended in DMEM with 10% FBS, 1% Pen/Strep, 25 mM HEPES, plated in culture flasks and incubated at 37°C, 5% CO_2_.

### Statistical analysis

One-way ANOVA followed by multiple comparison tests or t-tests were used for statistical analysis.

## Ethics compliance

All primary human cell lines used in this article were subject to IRB approval from the RI-MUHC REB (protocol: 2018–2922). All animal research in this article was subject to Facility Animal Care Committee approval from the RI-MUHC (protocol: 2001–6026).

## Results

### Absence of cytotoxicity in human fibroblasts

**Fig 2:**
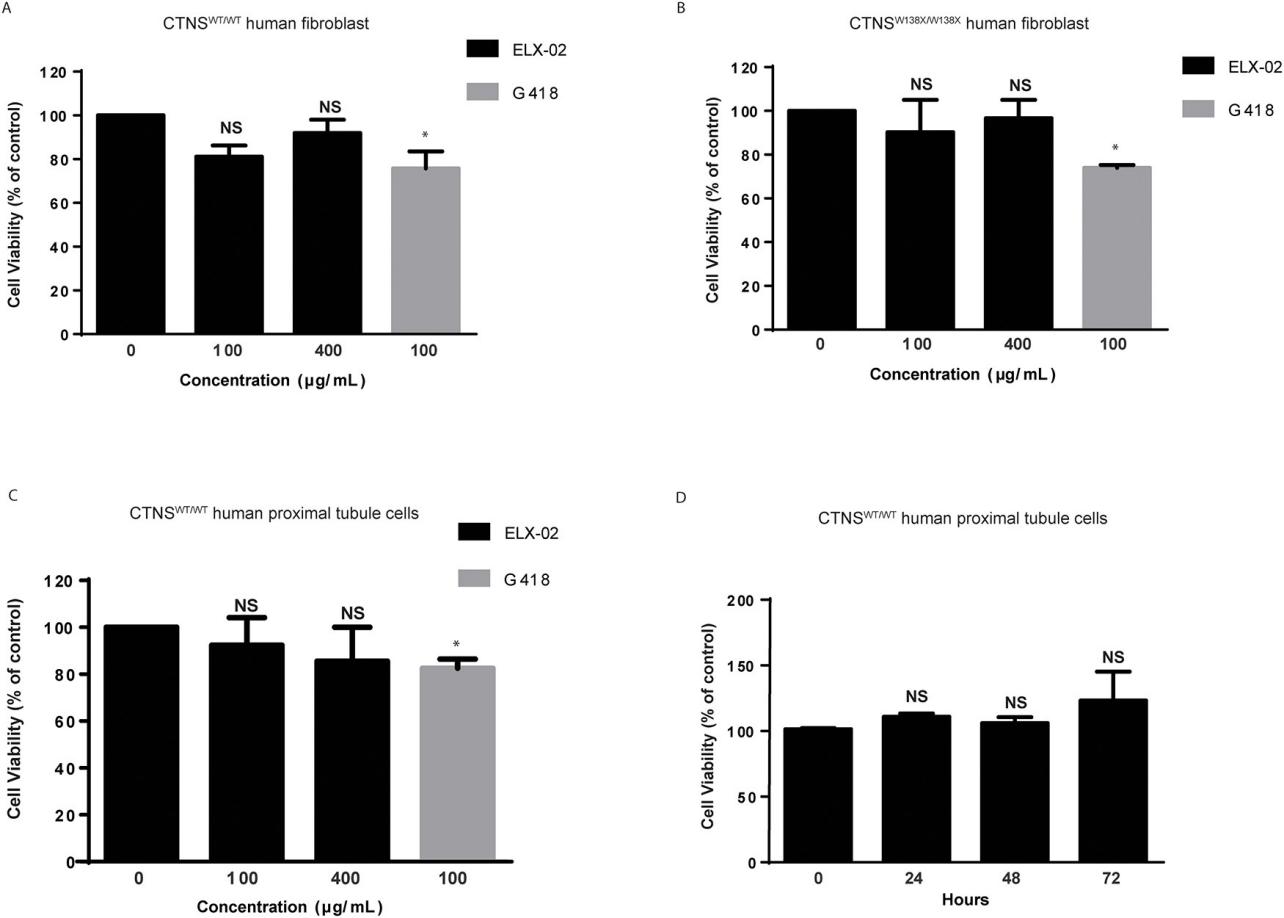
Cytotoxicity of ELX-02 in human fibroblasts and proximal tubule cells. **A**. Cytotoxicity assay in wildtype human fibroblasts (MCH070) showing no toxic effect of ELX-02 but cytotoxicity is observed with G418 after 48 h. **B**. Cytotoxicity assay in *CTNS*^*W138X/W138X*^ human fibroblast (WG1012) showing no toxic effect of ELX-02 but cytotoxicity of G418 after 48 h. **C**. Cytotoxicity assay in wildtype human proximal tubule cells (HK-2) showing no cytotoxic effect of ELX-02 but cytotoxicity with G418. **D**. Cytotoxicity assay in wildtype human proximal tubule cells (HK-2) showing no toxic effect of ELX-02 (400 μg/mL) at 0, 24, 48 and 72 h. (n = 4), One-way ANOVA and Dunnett’s multiple comparison test; (n = 4), t-test: * p<0.05.

To ascertain whether ELX-02 is toxic for human fibroblasts *in vitro*, we exposed monolayers of wildtype or homozygous *CTNS*^*W138X/W138X*^ mutant fibroblasts to ELX-02 (100, 400 μg/mL) or G418 (100 μg/mL) for 48 hours. Cell viability was assessed by LDH release into the culture medium and expressed as a percent of untreated controls. As seen in Fig 2A and 2B, cell viability was significantly reduced (to 76% of control, p<0.05) by G418 in both wildtype and mutant fibroblasts. In contrast, no significant toxicity was seen in the presence of ELX-02 at 100 or 400 μg/mL. Since aminoglycosides are concentrated by renal proximal tubular cells and exhibit clinical nephrotoxicity at high doses, we also assessed the toxicity of ELX-02 in a human proximal tubular cell line (HK-2). Whereas G418 (100 μg/mL) reduced HK-2 cell viability to 83% of untreated controls (Fig 2C), no reduction in cell viability was noted in the presence of ELX-02 (400 μg/mL) over 72 h (Fig 2D).

### Translational read-through of a *CTNS*^*W138X*^ mutant expression vector in human embryonic kidney (HEK293) cells and patient fibroblasts

**Fig 3:**
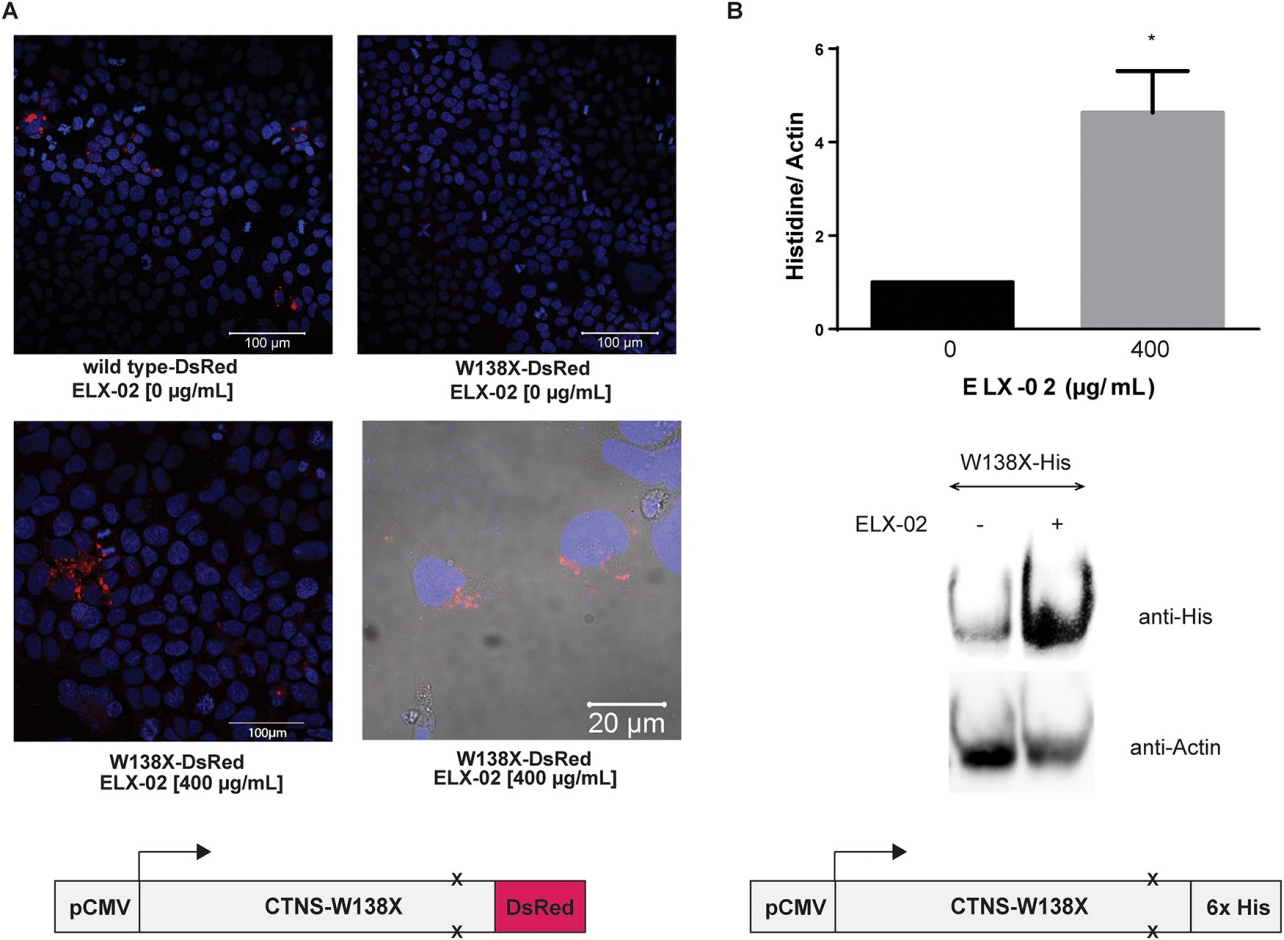
Effect of ELX-02 on exogenous CTNS protein expressed from p*CMV*-driven constructs. HEK293 cells transfected with expression plasmids containing a pCMV-driven *CTNS-DsRed* or *CTNS*^*W138X*^*-DsRed* fusion construct. **A**. (Top) Confocal images show CTNS-DsRed and W138X-DsRed fluorescence without ELX-02 treatment (400 X, scale bar = 100 μm); (middle) W138X-DsRed with treatment (400X, scale bar = 100μm) and a higher power confocal image showing intracellular expression of W138X-DsRed fusion protein following ELX-02 treatment (400 μg/mL) for 48h (1000 X, scale bar = 20 μm); (bottom) schematic diagram of plasmids. **B**. Immunoblot analysis of cells transfected with pCMV-*CTNS*^*W138X*^*-HIS* construct after ELX-02 treatment for 48 hours (400 μg/mL) (n = 4), two-tailed t-test: *p<0.05.

To ascertain whether ELX-02 induces translational read-through of the *CTNS*^*W138X*^ nonsense mutation, we transiently transfected HEK293 cells with a p*CMV*-driven expression vector containing either wildtype CTNS/DsRed or CTNS^W138X^/DsRed. After 48 hours, DsRed fluorescence was evident in cells transfected with wildtype but not the untreated nonsense mutant vector (Fig 3A). However, strong perinuclear expression of CTNS^W138X^/DsRed was induced by 48-hour exposure to ELX-02 (400 μg/mL). To confirm this observation, we also transfected *CTNS*^*del/del*^ fibroblasts (WG0881) with a *CTNS*^*W138X*^/*His* vector. As seen in Fig 3B, weak expression of the CTNS/His fusion protein was detectable on immunoblots probed with anti-His antibody. ELX-02 (400 μg/mL) treatment for 48 hours induced strong expression of CTNS^W138X^/His.

### ELX-02 induces translational read-through of endogenous *CTNS*^*W138X*^ in nonsense mutant fibroblasts

**Fig 4:**
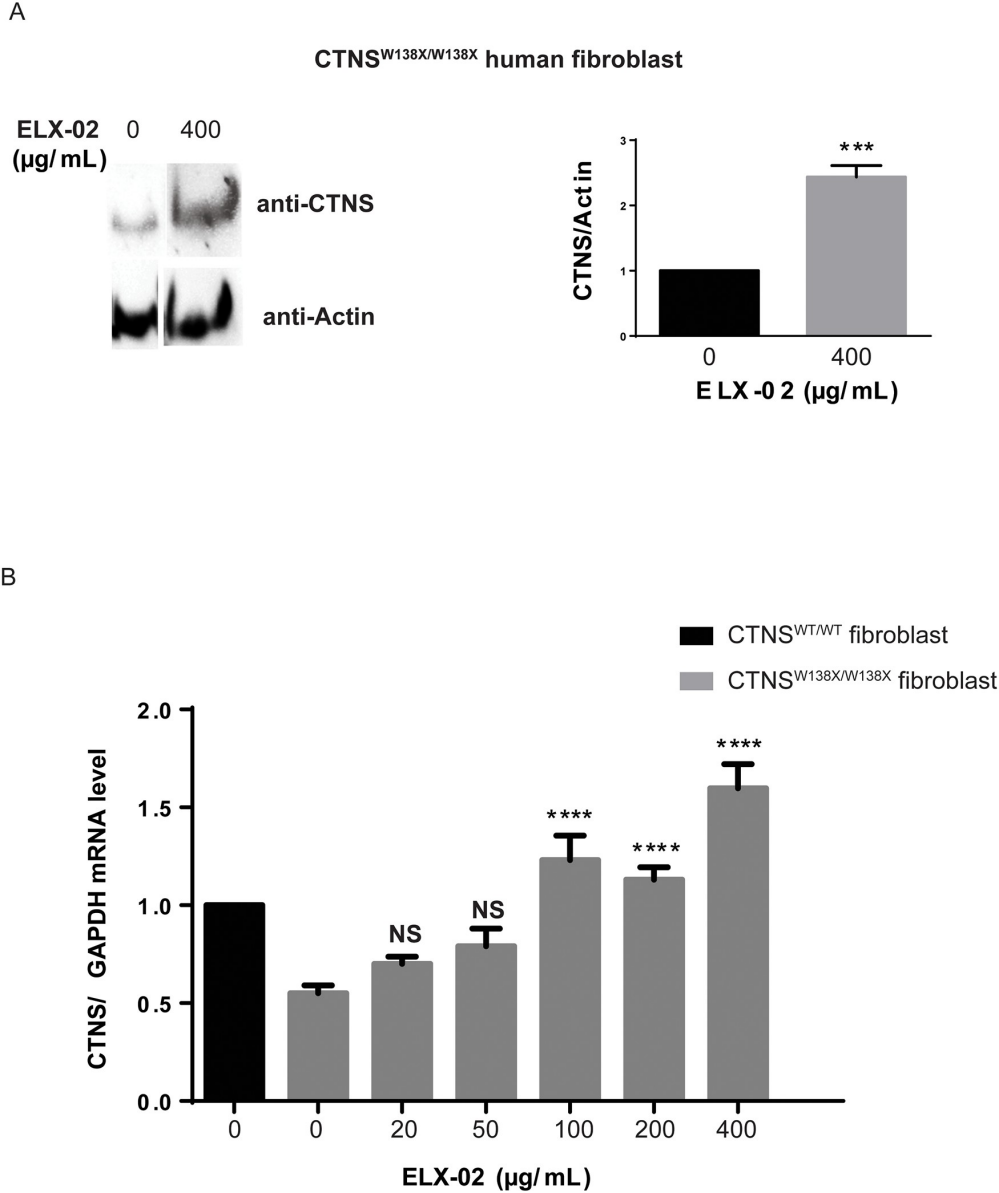
CTNS protein and mRNA levels in human fibroblasts. Wildtype (MCH070) and *CTNS*^*W138X/W138X*^ fibroblasts (WG1012) treated with ELX-02. **A**. Densitometry of immunoblots for CTNS in *CTNS*^*W138X/W138X*^ fibroblasts after 48-hour treatment with ELX-02 (400 μg/mL). **B**. RT-qPCR analysis showing increased mRNA transcript level in *CTNS*^*W138X/W138X*^ fibroblasts treated with ELX-02 at increasing concentrations (0, 20, 50, 100, 200 and 400 μg/mL) compared to untreated wildtype human fibroblasts (n = 4–8). One-way ANOVA and Dunnett’s multiple comparisons test or two-tailed t-test: **** p<0.0001, NS (not significantly different from untreated *CTNS*^*W138X/W138X*^ cells).

To confirm that ELX-02 permits expression of endogenous CTNS protein, we treated fibroblasts from a homozygous *CTNS*^*W138X/W138X*^ patient with ELX-02 (400 μg/mL) for 72 hours. As seen in Fig 4A, weak endogenous baseline CTNS expression was significantly increased by ELX-02 treatment. We also noted a dose-dependent (20–400 μg/mL) increase of ELX-02 on *CTNS* transcript level (Fig 4B). At ELX-02 concentrations of 100 μg/mL and above, *CTNS* transcript levels were greater that the normal transcript level of wildtype fibroblasts.

### ELX-02 induces expression of a functional CTNS protein

**Fig 5:**
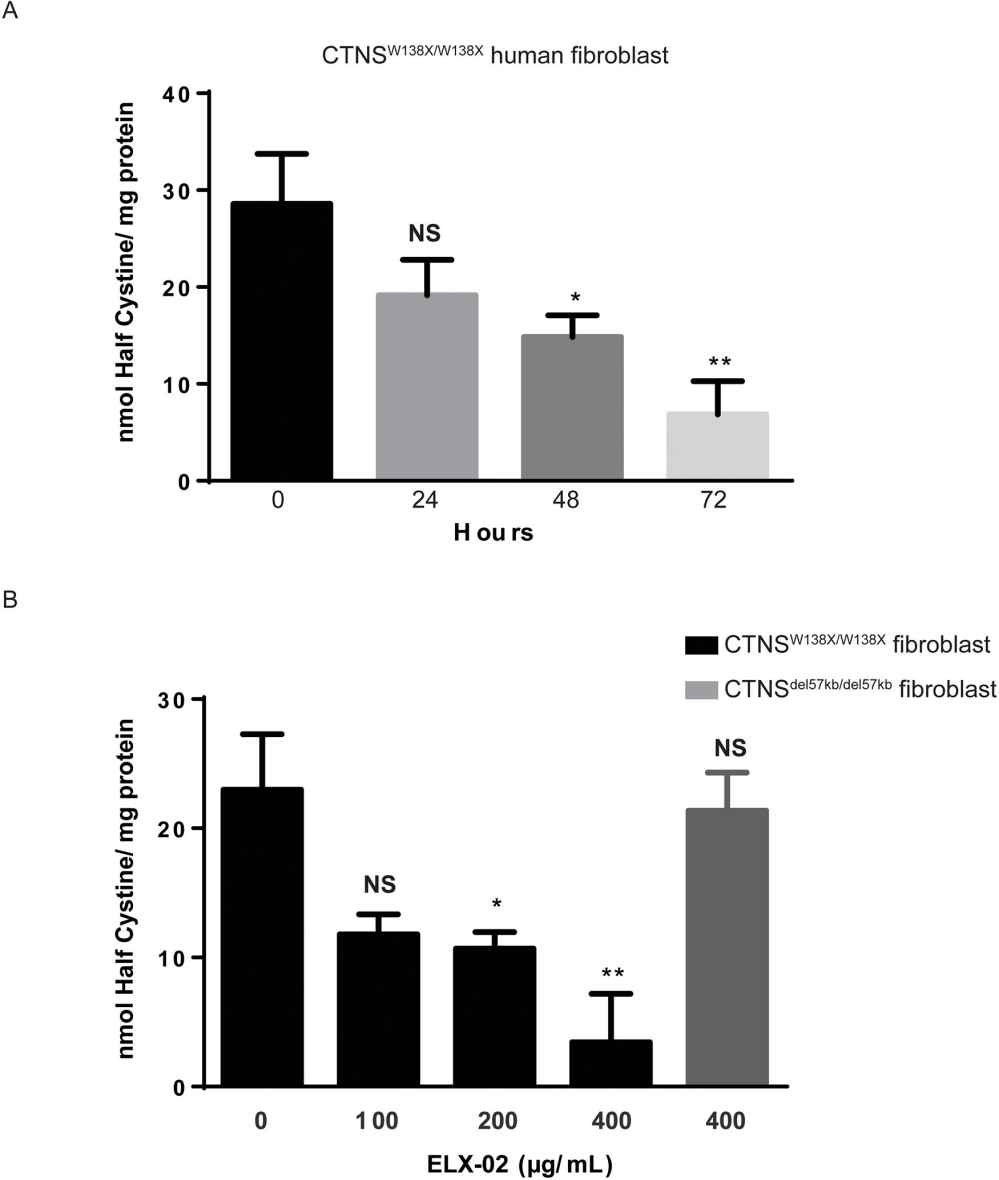
Effect of ELX-02 on pathological cystine accumulation in *CTNS*^*W138X/W138X*^ fibroblasts (WG1012). **A**. Half-cystine measurements after ELX-02 treatment (400 μg/mL) at various time-points (0, 24, 48 and 72 h) show significant decrease. **B**. Half-cystine measurements after 48 h of escalation doses of ELX-02 treatment (0, 100, 200 and 400 μg/mL, black bars) show decrease compare to ELX-02 treatment (400 μg/mL) in *CTNS*^*del57kb/del57kb*^ fibroblasts (WG0881) where it has no effect. (n = 4–8, grey bar), One-way ANOVA and Dunnett’s multiple comparison test: * p<0.05, ** p<0.01.

Fibroblasts from a *CTNS*^*W138X/W138X*^ cystinosis patient (WG1012) were treated with ELX-02 (400 μg/mL) for 72 h. As seen in Fig 5A, a time-dependent reduction in cellular cystine is observed; at 72 h, cystine level was 24% (±14% SEM) of untreated baseline. Cells were then treated with increasing concentrations of ELX-02 for 72 h. As seen in Fig 5B, ELX-02 induced a dose-dependent reduction of cystine in the nonsense mutant fibroblasts; at an ELX-02 concentration of 400 μg/mL, cell cystine was reduced to 15% (± 22% SEM) of untreated baseline. No effect of ELX-02 was noted on fibroblasts from a cystinosis patient harboring homozygous *CTNS*^*del/del*^ mutations.

### Effect of ELX-02 on nonsense mutant compound heterozygotes

**Fig 6:**
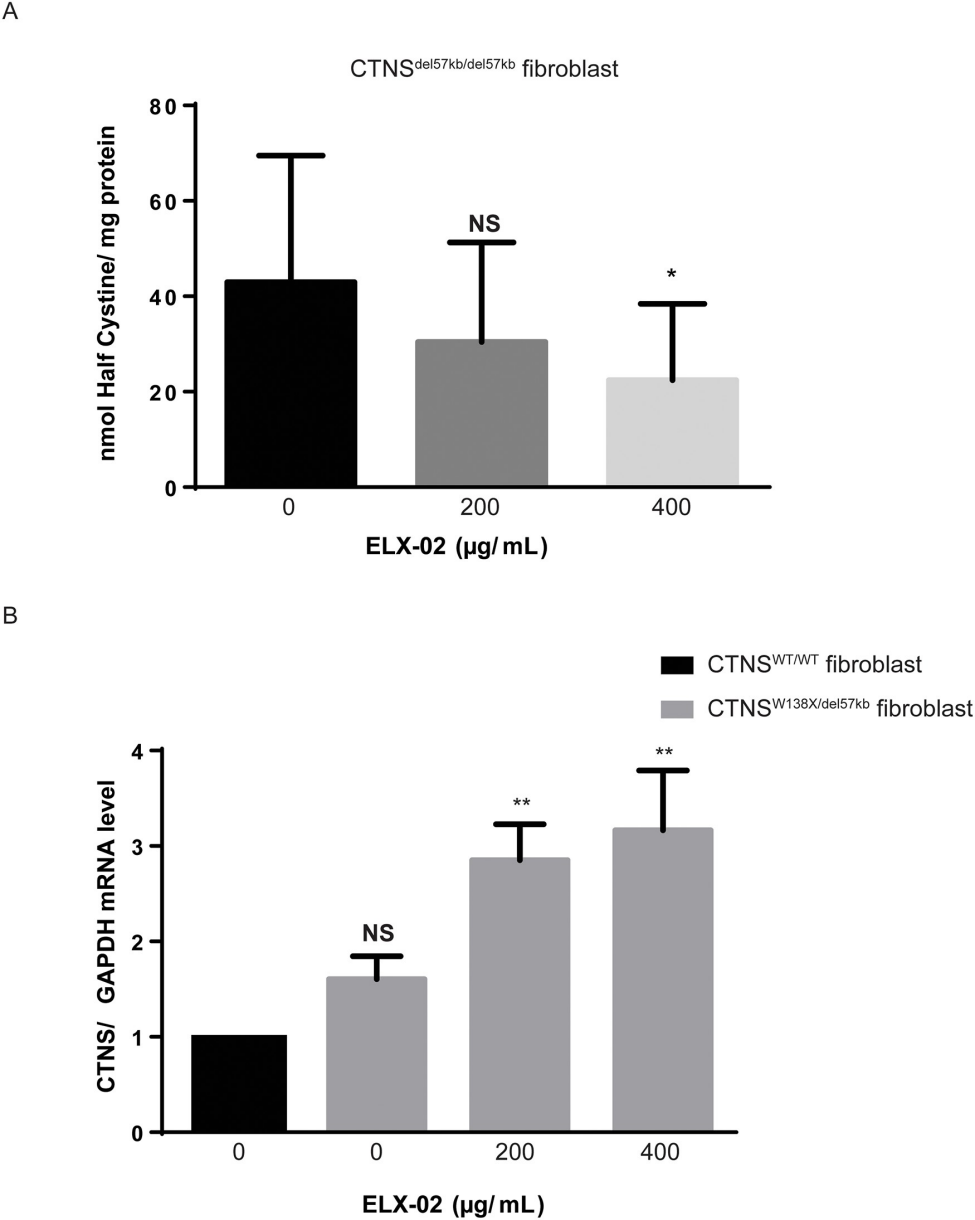
Effect of ELX-02 on pathologic cystine accumulation and mRNA levels in *CTNS*^*W138X/del57kb*^ fibroblasts (WG2379). **A**. Half-cystine per mg protein after 72-hour exposure to ELX-02 (200 and 400 μg/mL) was 53% of untreated baseline. **B**. CTNS transcript levels (RT-qPCR) were increased in *CTNS*^*W138X/del57kl*^ fibroblasts treated with ELX-02 (200 and 400 μg/mL) for 72 h, compared to *CTNS* wildtype fibroblasts, MCH070 (n = 3–4), One-way ANOVA and Dunnett’s multiple comparison test: * p<0.05, ** p<0.01.

Since the majority of cystinosis patients harbouring a nonsense mutation bear another class of mutation on the second *CTNS* allele, we studied the effect of ELX-02 on *CTNS*^*W138X/del57kb*^ fibroblasts (WG2379). As seen in Fig 6A, reduction of fibroblast cystine at 72 h (53% of untreated baseline) was similar to the reduction of cystine (62% of untreated baseline) seen in the homozygous *CTNS*^*W138X/W138X*^ mutant cells by that time (Fig 5A). As noted in homozygous nonsense mutant fibroblasts, ELX-02 restored *CTNS* transcript levels well above the normal level in wildtype cells (MCH070) (Fig 6B).

### Effect of ELX-02 in combination with cysteamine

**Fig 7:**
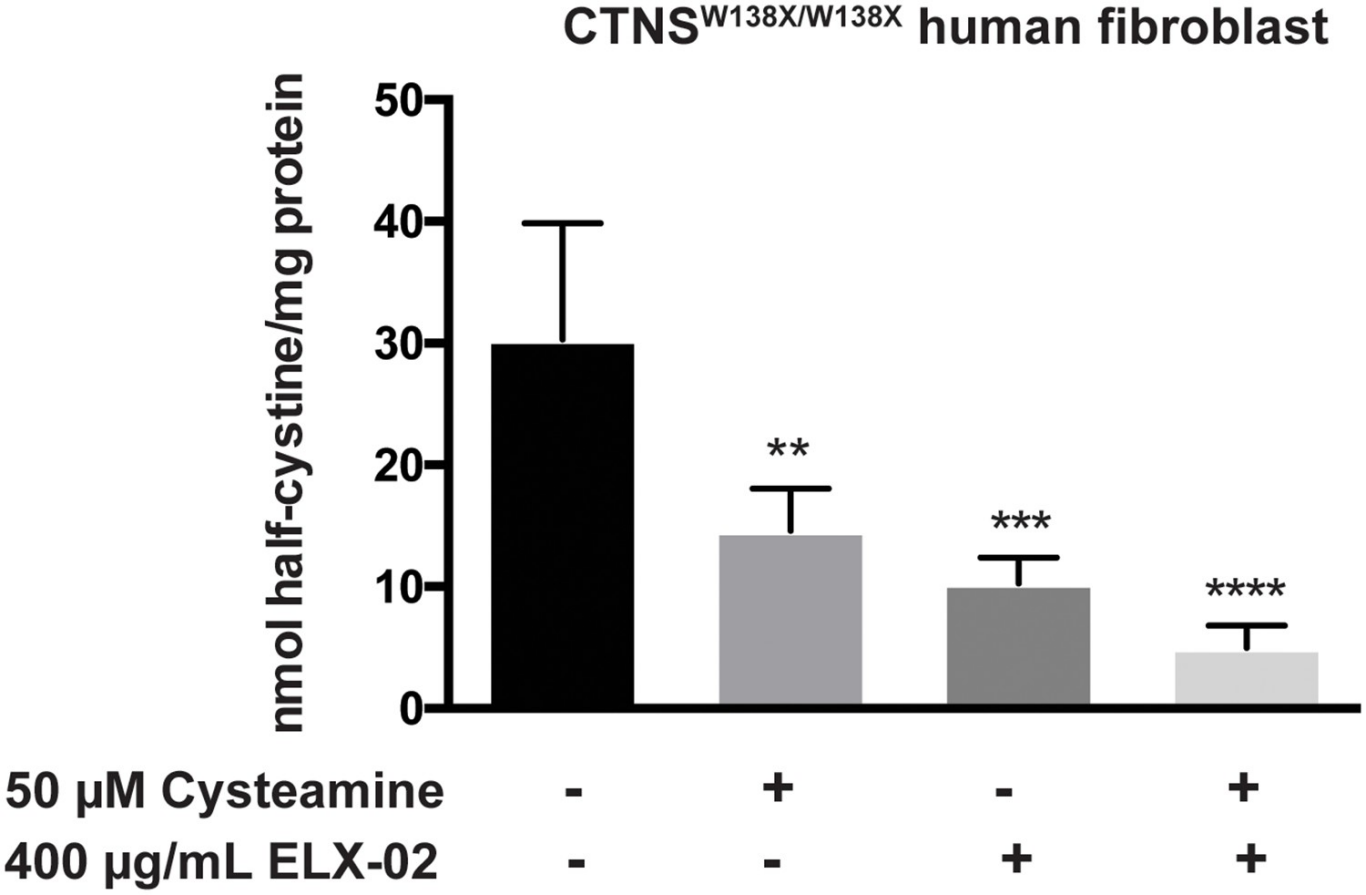
Effect of ELX-02 and cysteamine treatment on *CTNS*^*W138X/W138X*^ fibroblasts. Half-cystine was measured after 48 h exposure to ELX-02 (400 μg/mL) and cysteamine (50 μM). Both cysteamine and ELX-02 treatment alone reduce pathologic half-cystine accumulation, the combination therapy shows further reduction. (n = 3), One-way ANOVA and Dunnett’s multiple comparison test: ** p<0.01, *** p< 0.001 and **** p<0.0001.

Current therapy of cystinosis involves the cystine-depleting agent, cysteamine; oral doses (325 mg/m^2^ q6h) produce serum levels of cysteamine of about 50 μM [20]. Cysteamine forms mixed disulfides with intralysosomal cystine which can exit the lysosome via the PQLC2 channel [21]. We compared the effect of ELX-02(400 μg/mL) to the effect of cysteamine at the peak serum concentration achieved during standard oral therapy (50 μM) in *CTNS*^*W138X/W138X*^ fibroblasts (Fig 7). Each drug alone significantly reduced fibroblast cystine levels (60% and 50% respectively) compared to untreated baseline. Addition of ELX-02 to cysteamine appeared to have an additive effect, but reduction of cystine was not significantly different from either drug alone.

### *Ctns*^*Y226X/Y226X*^ nonsense mutation mouse model

**Fig 8:**
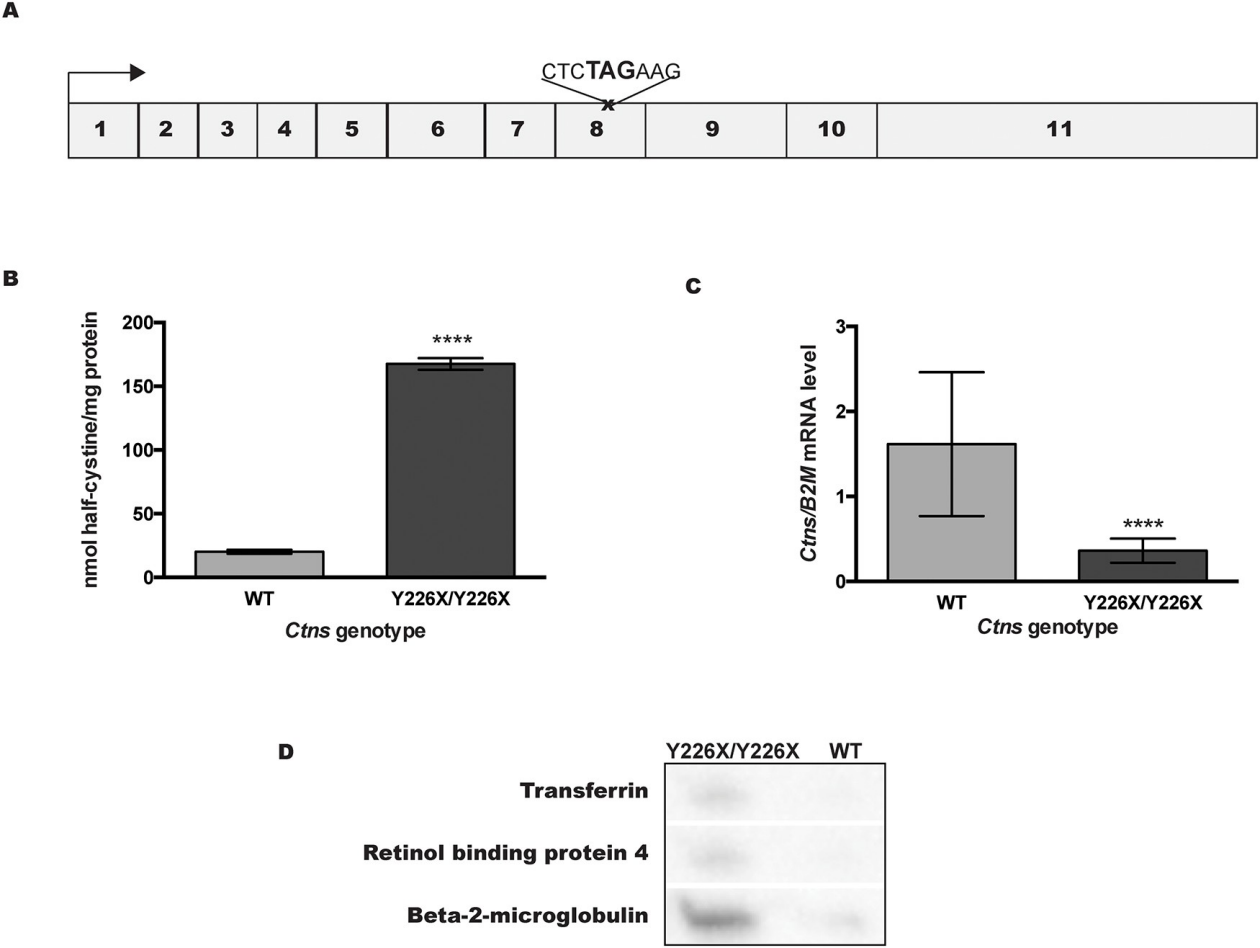
Characterization of the *Ctns*^*Y226X/Y226X*^ mouse model. **A**. Schematic diagram of *Ctns* transcript showing the *Ctns* Y226X nonsense mutation in exon 8. **B**. Half-cystine levels in 9-month old *Ctns*^*Y226X/Y226X*^ mouse kidneys was elevated compared to wildtype. **C**. *Ctns* transcript level (RT-qPCR) in *Ctns*^*Y226X/Y226X*^ mouse kidneys was reduced compared to wildtype. **D**. Urine levels of transferrin, retinol binding protein 4 and Beta-2-microglobulin (Western immunoblot) in 9 month-old *Ctns*^*Y226X/Y226X*^ mice were elevated, compared to wildtype. (n = 4), two-tailed t-test: ****p<0.0001.

To ascertain whether the nonsense mutation translational read-through effects of ELX-02 are evident *in vivo*, we generated a *Ctns* nonsense mutant mouse. Using a zinc-finger nuclease to edit the *Ctns* gene, we introduced a premature STOP codon (TAG) in *Ctns* exon 8 (Fig 8A). *Ctns*^*Y226X/Y226X*^ mice showed 8-fold increase in kidney cystine content (Fig 8B) and reduction of kidney *Ctns* transcript level to 22% of wildtype controls (Fig 8C). Prior to 6 months, the homozygous nonsense mutant mice appeared normal, but developed low-molecular-weight proteinuria by 9 months (Fig 8D).

**Fig 9:**
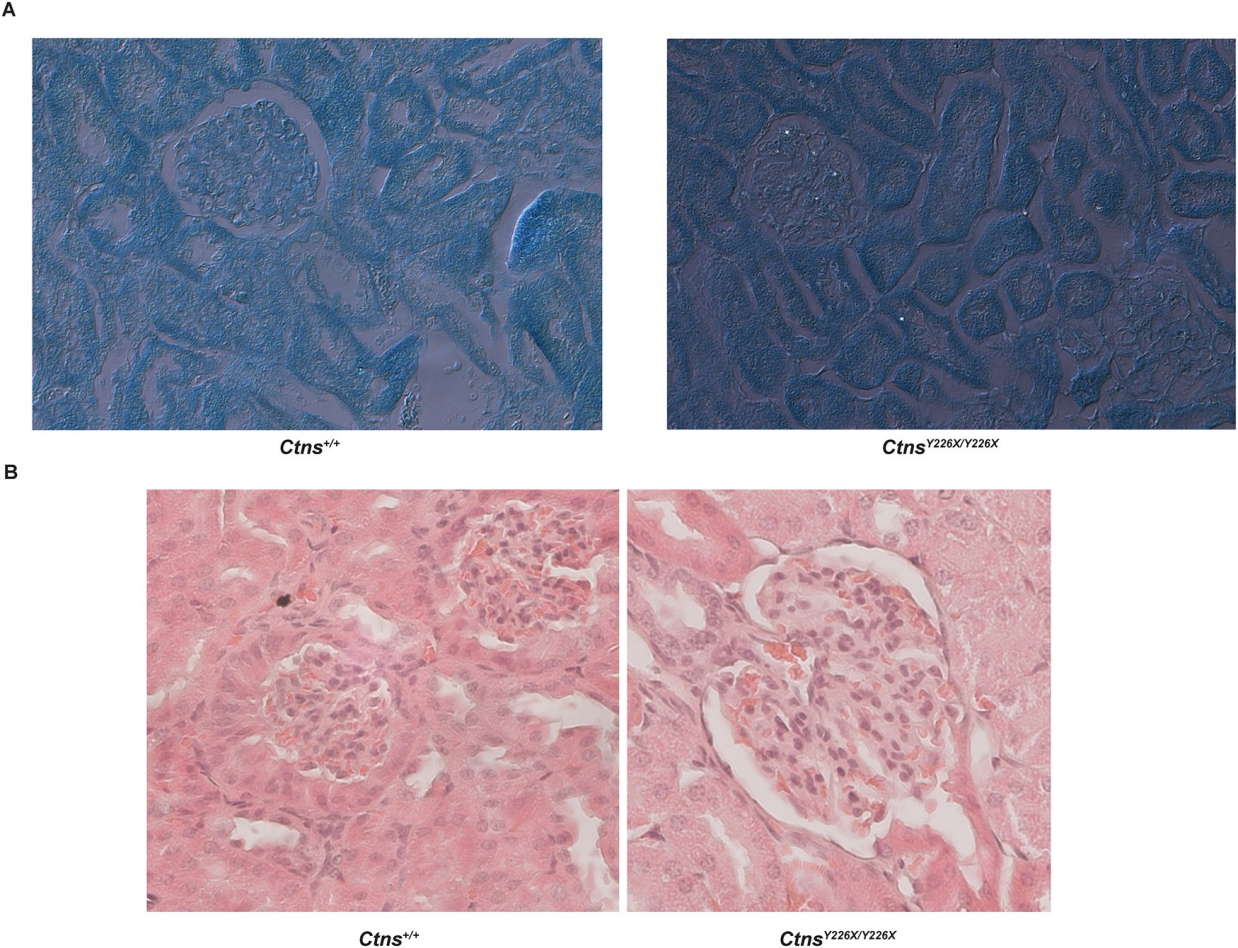
Histological features of kidneys from *Ctns*^*Y226X/Y226X*^ vs. *Ctns*^*+/+*^mice. **A**. Cystine crystals in 6-months old *Ctns*^*+/+*^ and *Ctns*^*Y226X/Y226X*^ female kidney. **B**. Bowman’s capsule/proximal tubule lesions in 6-month old *Ctns*^*Y226X/Y226X*^ male kidney vs *Ctns*^*+/+*^ wildtype.

Kidney sections from mutant mice showed scattered crystals under polarized light (Fig 9A) and occasional atrophy of columnar cells in Bowman’s capsule in male mice (Fig 9B).

### ELX-02 reduces cystine accumulation in embryonic fibroblasts derived from *Ctns*^*Y226X/Y226X*^ mice

**Fig 10:**
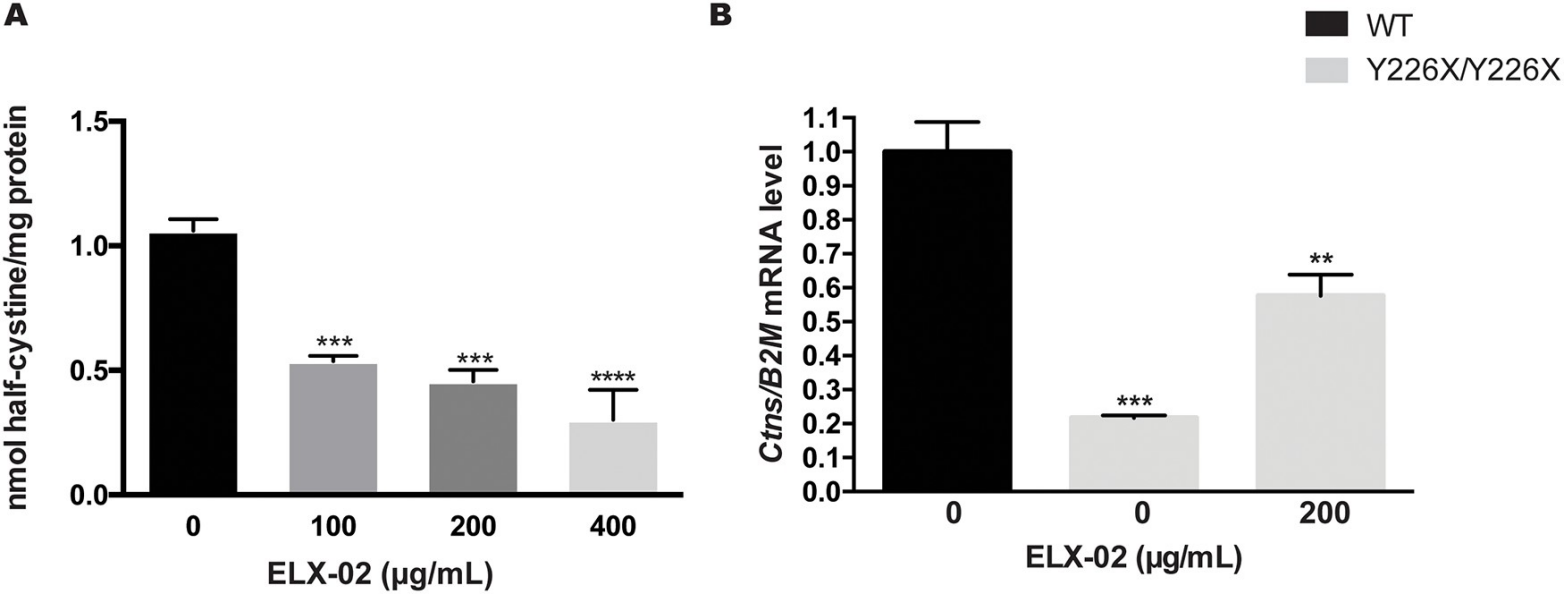
Effect of ELX-02 on cystine accumulation and CTNS transcription in embryonic fibroblasts derived from the *Ctns*^*Y226X/Y226X*^ mice. **A**. Half-cystine in MEFs from *Ctns*^*Y226X/Y226X*^ mice was progressively decreased by ELX-02 (0, 100, 200 and 400 μg/mL). **B**. *Ctns* transcript levels (RT-qPCR) in *Ctns*^*Y226X/Y226X*^ MEFs were increased by ELX-02 (200 μg/mL). (n = 3), One-way ANOVA and Dunnett’s multiple comparison test or two-tailed t-test: * p<0.05, ** p<0.01.

To confirm that ELX-02 induces sufficient *Ctns* read-through to reduce pathologic cystine accumulation in the Y226X nonsense mutant mouse, we isolated fibroblasts from mutant mouse embryos (MEFs), cultured them in monolayer and exposed the cells to various concentrations of ELX-02. ELX-02 had a dose-dependent effect on restoration of MEF *Ctns* transcript level (Fig 10A) and reduction of half-cystine (Fig 10B) at 48 h.

### Pharmacokinetics and tissue uptake of ELX-02 in *Ctns*^*Y226X/Y226X*^ mice

**Fig 11:**
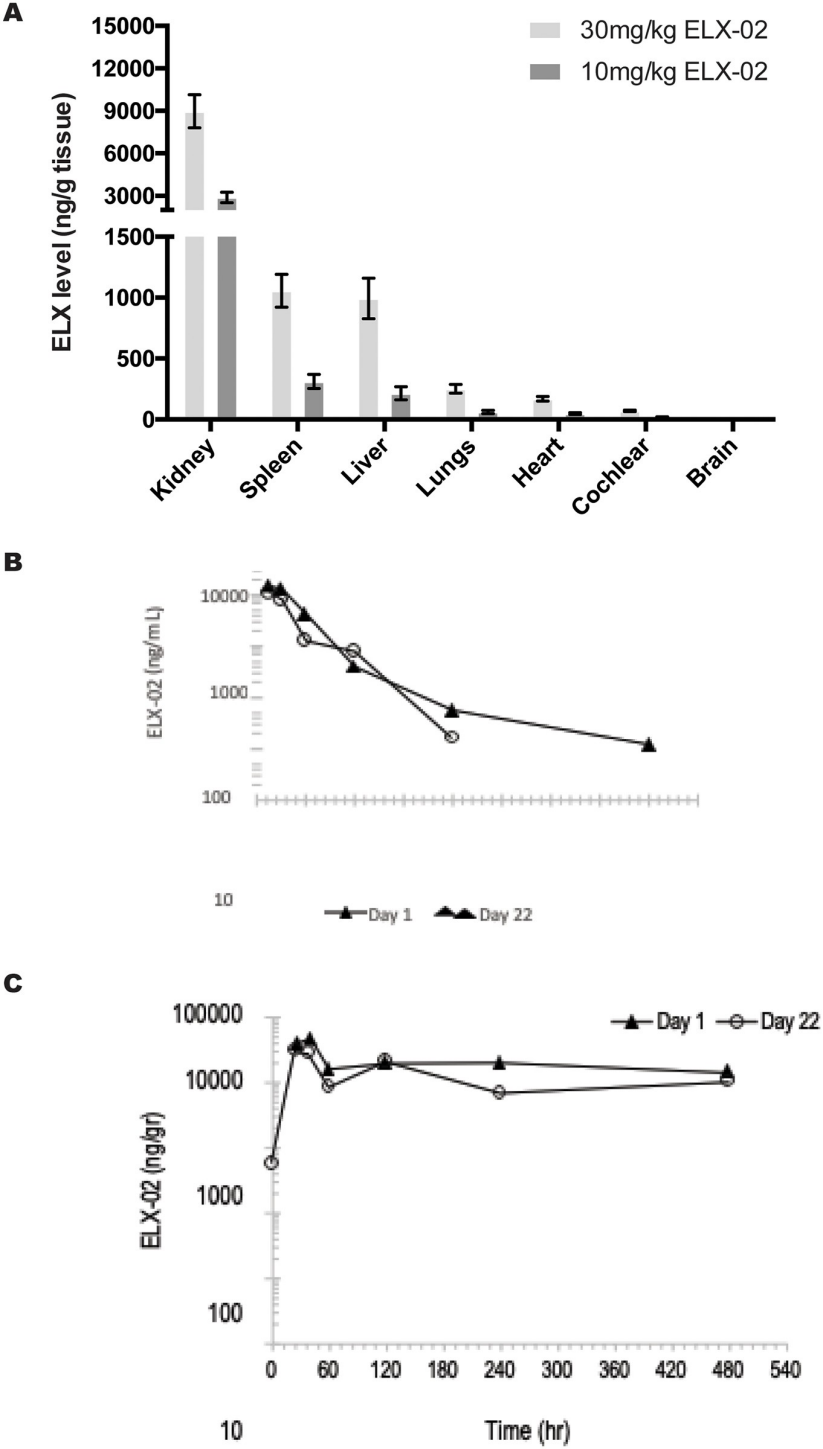
Measurement of ELX-02 drug accumulation in mouse tissues. **A**. Dose-dependent accumulation of ELX-02 in mouse tissues. **B**. Semi logarithmic plasma concentration vs. time following single and repeated subcutaneous administration of ELX-02 (10 mg/kg) to *Ctns*^*Y226X/Y226X*^ mice. **C**. Semi-logarithmic kidney concentration of drug vs time following single and repeated subcutaneous administration of ELX-02 (10 mg/kg) to *Ctns*^*Y226X/Y226X*^ mice.

ELX-02 levels in tissues of *Ctns*^*Y226X/Y226X*^ mice were measured following repeat subcutaneous administration (twice weekly, total of 8 doses) at dose levels of 10 and 30 mg/kg/dose. Highest levels of ELX-02 were measured in the kidney, followed by spleen and liver, with lower levels in other tissues (lung, heart, cochlea and brain) (Fig 11A). Accumulation in tissues was dose dependent without gender difference.

The pharmacokinetic profile of ELX-02 was assesed in plasma and kidney tissue of *Ctns*^*Y226X/Y226X*^ mice following single and repeat administration (twice weekly total of 7 doses) at a dosesof 10 mg/kg. In plasma ELX-02 was rapidly absorbed with a Tmax of 0.25 h after both single and repeated administration. ELX-02 was rapidly eliminated from plasma in a biphasic manner with the terminal half-life of 0.5 h. Systemic exposure (Cmax and AUC) following single and repeat dosing were similar (Fig 11B).

In the kidney Cmax was reached after 24–41 h and high levels were sustained up to 8 h post dose (Fig 11C). No gender differences were observed for any of the parameters in plasma and kidney.

### ELX-02 efficacy and tolerability in CtnsY226X/Y226X mice

**Fig 12:**
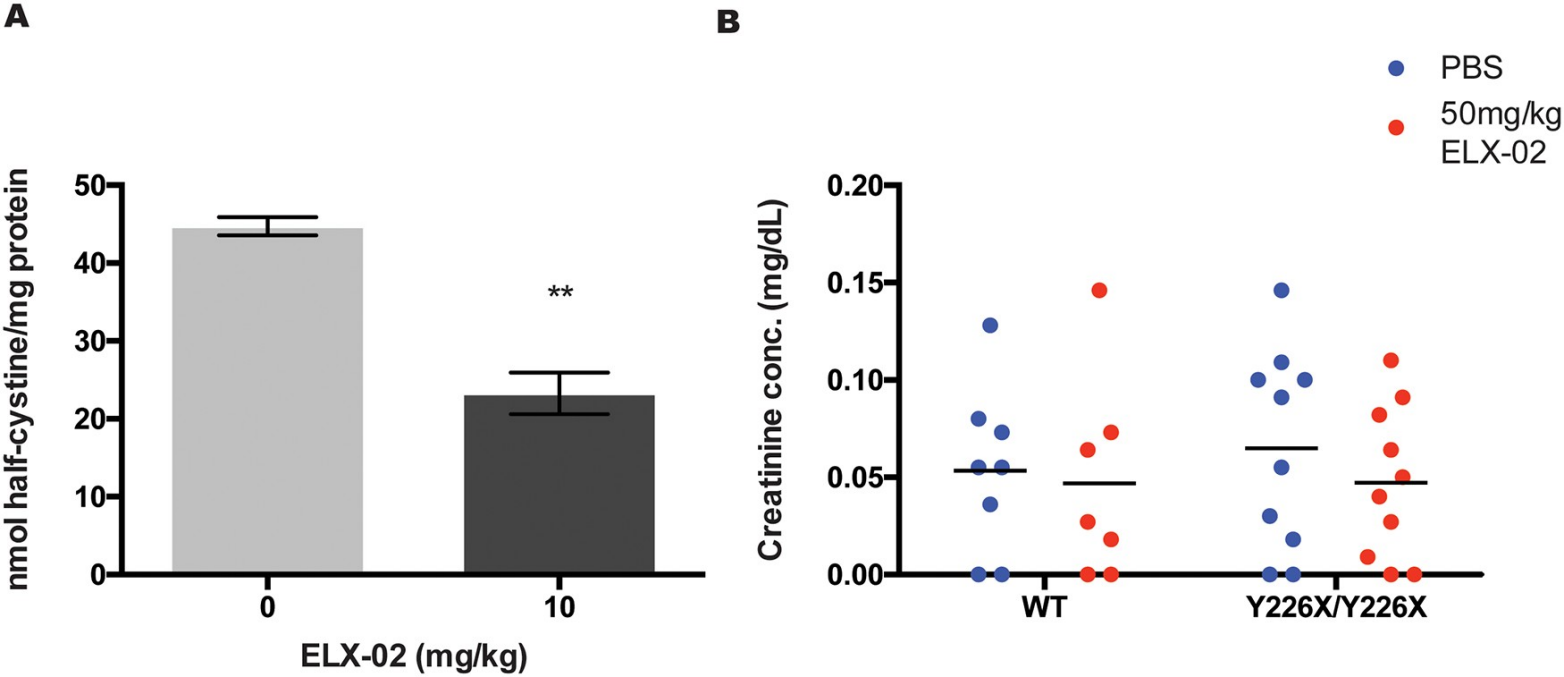
Effect of ELX-02 in *CTNS*^*Y226X/Y226X*^ mice. **A**. Half-cystine measurements show decrease pathologic accumulation in kidneys of *Ctns*^*Y226X/Y226X*^ mice treated with 7 injections of ELX-02 (10 mg/kg) over a 3-week period. **B**. Serum creatinine levels show no toxic effect of repeated ELX-02 treatment (50 mg/kg) compared to 1X PBS in both *Ctns* wildtype and *Ctns*^*Y226X/Y226X*^ mice. (n = 3), One-way ANOVA and Dunnett’s multiple comparison test or two-tailed t-test: * p<0.05, ** p<0.01.

The effect of ELX-02 on cystine levels was evaluated in *Ctns*^*Y226X/Y226X*^ mice, following repeated administration of ELX-02 (10 mg/kg/dose) or vehicle (Fig 12). ELX-02 reduced kidney cystine levels by 30% in treated animals compared with untreated animals (Fig 12A). No mice died, exhibited weight loss or changes in fur coat. Serum creatinine was similar in treated vs untreated animals (Fig 12B).

The *in vitro* effects of ELX-02 on reduction of cystine content in fibroblasts was evident within 24 h of treatment (Fig 4A). To predict how long the ELX-02-induced CTNS protein might last *in vivo*, we evaluated CTNS protein half-life in proximal tubule (HK-2) cells using a non-radioactive pulse-chase labeling assay[22]. CTNS protein was measured by Western blot and normalized to β-actin. Signal intensities were quantified using the Image Lab software (Bio-Rad).

**Fig 13:**
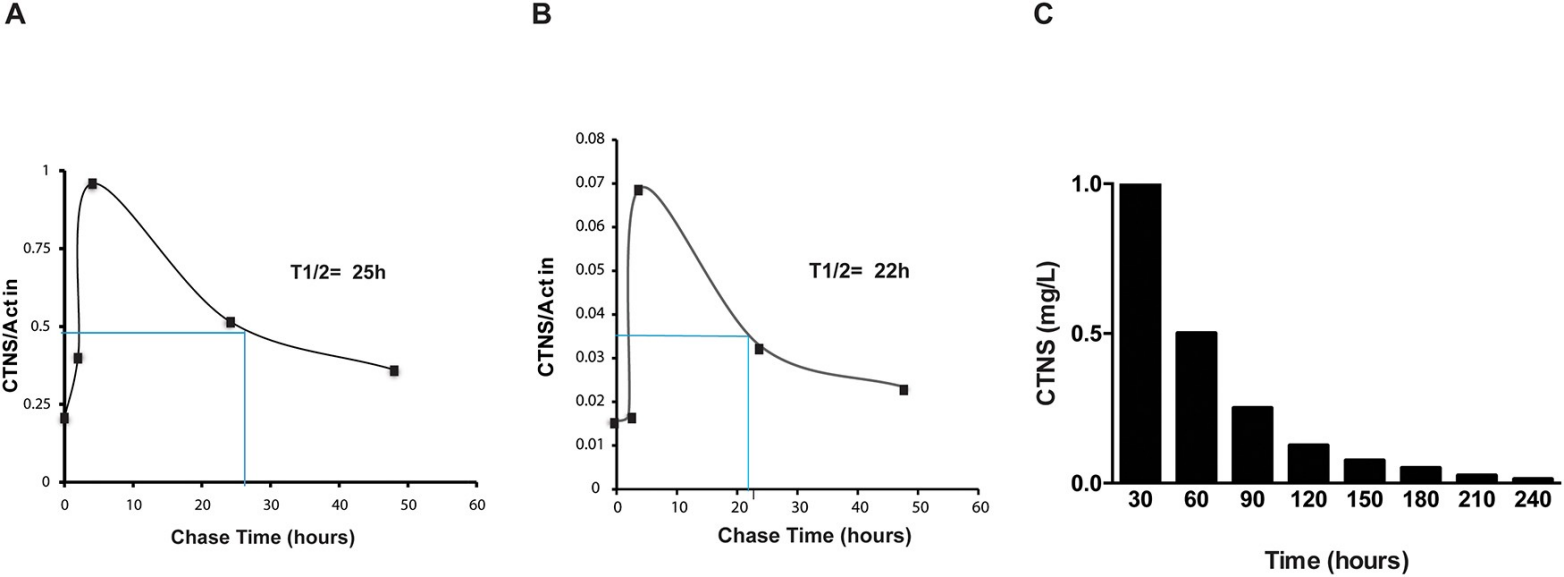
Cystinosin half-life estimation in a human proximal tubule (HK-2) cell line. HK-2 cells were pulsed in L-methionine-free medium with 50 μM of AHA at 2, 4, 6, 12, 24, and 48 h and then. Cells were then chased with L-methionine (2 mM). After biotinylation, streptavidin-precipitated proteins were analyzed by immunoblotting. **A**. anti-CTNS (N-epitope). **B**. anti-CTNS (C-epitope). The graph shows quantification of chemiluminescence signal at different time points. **C**. ProtLifePred server was used to predict cystinosin half-life. The calculation of CTNS half-life was predicted to be about 30 h.

Two representative examples of the exponential decay of cystinosin are shown in Fig 13, the quantification of CTNS protein half-life (t1/2) to be 25 h with anti-CTNS(N-epitope), 22 h with anti-CTNS(C-epitope). The calculation from 6 independent experiments revealed a CTNS half-life (mean ±SEM) of 24.89±3.69 hours. Furthermore, *in silica* algorithm based on molecular mass and N-end rule [23] was used to predict half-life of cystinosin (Fig 13). ProtLifePred server (http://protein-n-end-rule.leadhoster.com/) and ProtParam tool (https://web.expasy.org/protparam/) both give estimate cystinosin half-life of 30 h.

## Discussion

Our studies indicate that ELX-02 restores translational read-through of a functional full-length CTNS. Other investigators have shown that, by relaxing translational fidelity, aminoglycosides permit binding to a near-cognate transfer RNA at the premature STOP codon and most often inserts the wildtype amino acid [13, 24]. Our data demonstrate that ELX-02 induces read-through of an exogenous *CTNS*^*W138X*^ construct and of the endogenous *CTNS*^*W138X*^ in patient fibroblasts. Furthermore, the magnitude of the ELX-02 (200–400 μg/mL) effect is sufficient to suppress nonsense mutation-induced mRNA decay and reverse pathologic accumulation of cystine. As seen in Fig 7, ELX-02 reduced fibroblast cystine to levels similar to those achieved by cysteamine *in vitro* (at peak blood levels reported in patients) [20] and *in vivo* during standard clinical therapy [5]. This benefit is achieved by nonsense mutation read-through, since no effect of ELX-02 is seen in fibroblasts harboring a biallelic *CTNS* deletion.

The leukocyte cystine level of healthy heterozygous mutant *CTNS* carriers is only marginally higher than that of normal individuals [25], yet *in vitro* cystine efflux rate from isolated patient lysosomes is about 50% of normal [26]. Thus, rescue of CTNS function to 50% of normal correlates with clinical benefit. Kalatzis *et al* estimated that lysosomal cystine efflux rates are about 19% (±10%) of normal in patients with adult-onset ocular cystinosis and about 9–20% in patients with juvenile onset ocular disease [27]. Therefore, the threshold for clinical benefit seems to lie between 20% and 50% residual activity. Our studies did not directly measure the effect of ELX-02 on lysosomal cystine efflux rate, but since cell cystine is significantly reduced, efflux must be at least sufficient to exceed the rate at which cystine is generated within lysosomes.

Current therapy of cystinosis involves oral cysteamine (1.3g/m^2^/day) in four divided doses. At this dose, peak serum cysteamine is about 50 μM [20]. Oral cysteamine delays renal transplantation, improves growth and reduces hypothyroidism in cystinosis [5]. In our study, homozygous *CTNS*^*W138X*^ fibroblasts exposed to 400 μg/mL ELX-02 for 48 h achieved reduction of cell cystine to 33% of untreated baseline; this was slightly better than the effect of 50 μM cysteamine (50% of untreated baseline) for 48 h. Thus, the magnitude of the ELX-02 effect on homozygous *CTNS*^*W138X/W138X*^ cells appears to be clinically relevant. Interestingly, ELX-02 was only slightly less effective in cells where the W138X mutation was in compound heterozygosity with a *CTNS* deletion allele. This is important, since the majority of cystinosis patients are not likely to have biallelic nonsense mutations. At least six other CTNS nonsense mutations have been reported and it is likely that the efficacy of ELX-02 varies somewhat, depending on the specific mutation and its nearby sequence context [24, 28, 29] as in other genetic diseases (Table 1).

To extend our observations, we used zinc-finger nuclease editing to generate a *Ctns*^*Y226X*^ nonsense mutant mouse model. The mutation was inserted into *Ctns* exon 8, to truncate the protein at a position similar to the *Ctns*^*neo/neo*^C57Bl6 knockout mouse [19]. On the CD1 background, the renal phenotype of *Ctns*^*Y226X*^ mice was somewhat milder than that of *Ctns*^*neo/neo*^*/C57Bl6* knockout mice, but kidney cystine was elevated, *Ctns* transcript was reduced and cystinosin protein was undetectable in mutant kidney. Between 6–9 months, mutant mice developed low molecular weight proteinuria and scattered atrophy of proximal tubules (“swan neck deformity”) with bi-refringent crystals. When the mice were infused with a single dose of ELX-02 (10 mg/kg), plasma levels fell rapidly but the drug was concentrated in kidney 10–15 times the peak level in blood and displayed a prolonged retention phase over 2–3 days. This corresponds to the characteristic uptake and sustained retention of aminoglycosides in association with intracellular megalin, reported by others [30]. In the HK-2 proximal tubular cell line, we estimated the half-life of cystinosin protein to be about 22–30 h. On the basis of these observations, we infused 6-month old *Ctns*^*Y226X/Y226X*^ mice with 7 repeated intravenous doses of ELX-02 (10 mg/kg) twice per week and found that kidney cystine was significantly reduced. Thus, ELX-02 displays favorable kidney pharmacokinetics for intermittent dosing and significantly reverses pathologic renal accumulation of cystine *in vivo* without short-term nephrotoxicity (elevation in serum creatinine).

Although the *CTNS*^*W138X*^ allele is prevalent (40–50%) among French Canadian cystinosis patients, it is distributed across North America and Europe at lower frequency [31]. Other CTNS nonsense mutations have been reported but these are uncommon and, in their seminal report, Town *et al* found that about 15% of cystinosis patients worldwide carried one or more *CTNS* nonsense mutations [27, 32]. Thus, the potential of aminoglycoside read-through therapy would be limited to a specific subset of cystinosis patients. However, if it works in cystinosis, there is no *a priori* reason why the same strategy cannot be applied to any of the >4000 known genes with phenotype-causing mutations (OMIM).
